# Targeting STING Signalling Polarizes Tumor-Associated Neutrophils to Boost Anticancer and Abscopal Antimetastatic Functions

**DOI:** 10.2147/ITT.S576457

**Published:** 2026-02-19

**Authors:** Alina Drzyzga, Justyna Czapla, Tomasz Cichoń, Sybilla Matuszczak, Ewelina Pilny, Magdalena Jarosz-Biej, Joanna Ciepła, Ryszard Smolarczyk

**Affiliations:** 1 Center for Translational Research and Molecular Biology of Cancer, Maria Skłodowska-Curie National Research Institute of Oncology, Gliwice Branch, Gliwice, 44-102, Poland

**Keywords:** stimulator of interferon genes, STING, STING agonist, tumor-associated neutrophils, TANs, cancer immunotherapy, metastasis

## Abstract

**Introduction:**

The stimulator of interferon genes (STING) pathway has emerged as a promising target for cancer immunotherapy. Tumor-associated neutrophils (TANs) are underrated among the cells involved in STING-targeted therapies. The polarization state of TANs divides them into antitumor N1 and tumor-supportive N2 TANs. Our purpose was to assess whether STING activation stimulates the repolarization of TANs toward the N1 subtype and to investigate the potential participation of TANs in metastasis and macrophage polarization processes.

**Methods:**

This research was carried out on murine B16-F10 melanoma and 4T1 breast carcinomas. Flow cytometry, immunofluorescence microscopy, and multiplex immunoassay were used to assess the phenotype of TANs. Magnetic cell sorting was employed to assess the cytotoxic potential of TANs. The spontaneous 4T1 lung metastasis model enabled assessment of the impact of primary tumor targeting with STING agonist on the inhibition of metastasis and lung immune landscape changes. To verify the influence of in vivo cGAMP-activated TANs on the macrophages phenotype, the co-culture experiments of bone marrow-derived macrophages (BMDM) and TANs were conducted.

**Results:**

In both types of tumors, STING targeting converted TANs polarization state toward anticancer N1 subtype, marked by an increase in ICAM-1, CD69, Fas, CXCL10, TNFα, CCL3 expression, and a decrease in VEGF. Targeting STING induced a cytotoxicity of TANs against 4T1 cancer cells, while neutrophils masking revealed their importance for the maintenance of health conditions. Treatment of primary 4T1 tumors resulted in inhibition of metastasis and conversion of lung immune cells toward an anticancer phenotype. Direct co-cultivation of TANs derived from cGAMP-treated tumors with BMDM polarized them toward M1-like cells.

**Conclusion:**

Targeting STING provides polarization of TANs phenotype toward anticancer N1 subtype. Obtained data fills the gaps in the knowledge of the impact of STING-activating therapy on TANs functions, participation in the metastasis process, and acquisition of M1 phenotype by macrophages.

## Plain Language Summary

Immunotherapy emerges as a key modality to fight cancers. Among such therapies, activating the STING pathway enables immune cells to recognize and destroy the tumor. In this study, we focused on one of the most abundant immune cell populations in tumors called tumor-associated neutrophils (TANs). We found that using STING activator enables TANs to switch toward antitumor cells. We also showed that such activated cells participate in the shift of another immune cell, called macrophages, into antitumor cells, and that STING activator provides an antimetastatic effect.

## Graphical Abstract

**Figure:**
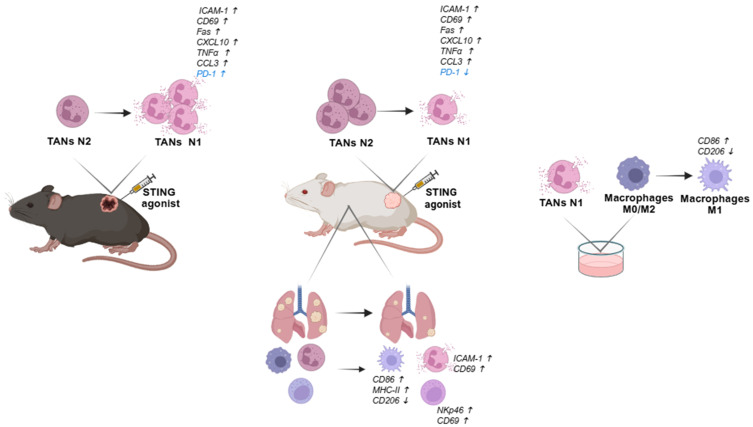

## Introduction

Stimulator of interferon genes (STING) plays an essential role in activating innate and specific immunity in novel cancer immunotherapies. Its activation with proper agonists, eg, natural STING ligand – cGAMP, leads to the production of multiple proinflammatory cytokines and type I interferons (IFNs), enabling conversion of the “cold” tumor microenvironment into the “hot” one. This occurs based on immune cells infiltration and their polarization toward the antitumor phenotype. The potent activation of immune cells is a key step in destroying cancer cells, providing an efficient and long-lasting immune response.^1^,^2^ STING activation may induce cell death, leading to the release of cancer antigens and enabling their presentation. Further, through induction of specific chemokine expression and endothelial cells’ STING signalling, the trafficking and infiltration of T cells toward tumors is facilitated.^3^ Importantly, STING activation is known to upregulate PD-L1 expression. Its induction decreases the antitumor effects, thus multiple approaches are undertaken to evade this mechanism.^4^ Based on complex preclinical data, the currently ongoing clinical trials focus on combination of STING agonist with clinically approved PD-1/PD-L1 inhibitors (eg Pembrolizumab, Nivolumab, Atezolizumab).^5^

The regulation of STING results in multiple changes in the state of specific immune cells. Among them, the transition of the tumor-associated neutrophils (TANs) phenotype and the subsequent consequences need further investigation and should be taken into consideration when planning STING targeting therapies. TANs constitute a heterogeneous population of cells that, depending on the circumstances, may exert either pro- or antitumor activities. Their antitumor mode of action depends on diverse mechanisms, such as reactive oxygen species (ROS) production, granules release, and induction of recruitment and activation of cytotoxic CD8^+^ T cells.^6–8^ On the other hand, despite the short life span of neutrophils, their abundance within tumors is a predictor of cancer progression and poor clinical outcomes.^9^,^10^ TANs may promote cancer cells proliferation, angiogenesis, and metastasis.^6^,^11^,^12^ Therefore, new approaches are being proposed to tackle and overcome TANs-mediated unfavourable effects and boost their antitumor potency. Currently, it is well proven that blocking TGF-β or treatment with IFN-β changes the TANs phenotype from protumor toward antitumor one. Depending on the manifested phenotype, TANs are classified as N1 antitumor cells and N2 protumor cells.^13^,^14^ The N1 TANs phenotype is distinguished by the following markers: ICAM-1^high^, Fas^high^ (CD95), iNOS^high^, high TNFα, CXCL10 (IP-10), CCL3, ROS production, and decreased VEGF level.^14–18^ The tumor-supportive effects triggered by neutrophils are directly related to their plasticity. The metastasis process, one of the hallmarks of cancer, is partially driven by the function of neutrophils.^19–21^ Moreover, TANs have been shown to modulate other immune cells to create an immunosuppressive tumor microenvironment.^22^,^23^

STING activation stimulates the production of TANs-polarizing agent IFN-β. Thus, we aimed to assess STING agonist-induced repolarization of the TANs phenotype in two murine tumor models with diversified STING status: STING^high^ melanoma B16-F10 and STING^low^ breast carcinoma 4T1. In this study, we determined activation, infiltration, and polarization of TANs following in vivo STING agonist administration. Furthermore, we assessed STING agonist-induced TANs cytotoxicity in a murine breast carcinoma model. Additionally, we examined the STING agonist-induced switch of the lung immune cells phenotype in a murine model of spontaneous breast carcinoma metastasis. Finally, the impact of STING-induced TANs on the macrophage phenotype was investigated.

It was found that in both types of tumors, the STING agonist induced conversion of the TANs phenotype toward N1-like. In the breast carcinoma model, we confirmed the STING agonist-induced cytotoxicity of TANs against cancer cells and the ability of STING agonist-activated TANs to convert macrophages into M1 antitumor cells. Finally, we demonstrated the potential of the STING agonist to enhance the abscopal effect, which is associated with the activation and polarization of multiple immune cells at the metastatic site.

## Materials and Methods

### Mice and Ethics Statement

Experiments were conducted on female BALB/c and C57Bl/6NCrl mice (8–10 weeks old) obtained from Charles River Breeding Laboratories (Wilmington, MA, USA). Animals were treated in accordance with the recommendations regulated by European and national laws: Directive 2010/63/EU of the European Parliament and consistent with it Polish Act on the protection of animals used for scientific or educational purposes (Journal of Laws 2015, item 266), amending the minimum requirements to be met by a center and the minimum requirements for the care of animals. Experiments on animals were conducted in accordance with the 3R rules with the consent of the Local Ethical Committee for Experiments on Animals at the Medical University of Silesia in Katowice (decisions No. 49/2021 and 26/2024). The study adhered to the ARRIVE guidelines. Mice were housed in the Maria Skłodowska-Curie National Research Institute of Oncology, Gliwice Branch (Poland) in a pathogen-free facility (SPF standard) in a HEPA-filtered Allentown’s IVC System. The mice received a total pathogen-free standard and complete diet (Altromin International, Lage, Germany) and water ad libitum. The cages were equipped with enrichment elements (cardboard houses, nesting material). Sample size was estimated using a one-way ANOVA test; at least 2 independent experiments were performed, n=4-5 mice per group, per experiment, total n=250 of BALB/c mice and n=150 of C57Bl/6NCrl mice. Murine B16-F10 and 4T1 cells were injected subcutaneously (2×10^5^ cells, lower-flank injection). If not indicated otherwise, cGAMP/PBS was administered on the 14^th^ day post cancer cells inoculation. The animals were euthanized following the ethical guidelines for animal experimentation by cervical dislocation 12 hours following cGAMP/PBS administration. Anaesthetic agents were used as described in the specific methods.

### Cell Lines and Mice Therapeutic Agents

Murine melanoma B16-F10 and breast carcinoma 4T1 cell lines (ATCC, Manassas, VA, USA) were cultured under standard conditions (37°C, 5% CO_2_, and 95% humidity) in RPMI 1640 medium (Biowest, Nuaillé, France) supplemented with 10% fetal bovine serum (FBS’ EURx, Gdansk, Poland) and 1% penicillin–streptomycin (Biowest). 2′3′-cGAMP VacciGrade™ (cGAMP, InvivoGen, Toulouse, France) was administered by intratumoral injection at a dose of 5μg/100μL PBS^−^/mice. Mice in the control groups received 100μL PBS^−^.^24^ Mouse anti-Ly6G IgG2a, Kappa (clone: 1A8, AbsoluteAntibody, Oxford, UK), mouse isotype control IgG2a, Kappa (AbsoluteAntibody) antibodies (used for neutrophil depletion) were administered intraperitoneally at a dose of 100 μg/mouse, acc. to Olofsen et al.^25^ Luminol sodium salt (Santa Cruz Biotechnology, Dallas, TX, USA) was administered intraperitoneally at a dose of 6 mg per mouse.

### Flow Cytometry Assessment

Tumors were digested with collagenase II solution (500 U/mL; Gibco™, Thermo Fisher Scientific, Waltham, MA, USA). Mononuclear cells and granulocytes were collected using a Histopaque-1119 gradient (Merck). Cells were blocked with anti-mouse CD16/32 (clone: 93) antibody (BioLegend, San Diego, CA, USA) and stained with the following antibodies (BioLegend): anti-CD11b (clone: M1/70, FITC), anti-Ly6G (clone: 1A8, APC-Cy7), anti-CD69 (clone: H1.2F3, PE-Cy7), anti-PD-1 (clone: 29F.1A12 PE-Cy7), anti-PD-L1 (clone: 10F.9G2 PE-Cy7), anti-CD54 (ICAM1, clone: YN1/1.7.4, APC), anti-Fas (clone: SA367H8, APC), anti-FasL (clone: MFL3, APC), anti-MMP9 antibody (Merc, AlexaFluor488). Dead cells were stained using DAPI (Merck). Gates dividing negative from positive cells were based on isotype control (BD FACSCanto, Becton Dickinson, Franklin Lakes, NJ, USA).

### Confocal Microscope Analysis

Tumors were collected and frozen in liquid nitrogen. The frozen sections (5 µm) were examined with anti-Ly6G antibody conjugated with AlexaFluor594 (BioLegend) and anti-MMP-9 antibody (polyclonal, Merck, Darmstadt, Germany), followed by FITC-conjugated secondary antibody (Vector Laboratories; Burlingame, CA, USA). Tumor sections were counterstained with DAPI (Merck). Sorted TANs were cytospined, stained with anti-Ly6G antibody coupled with AlexaFluor594 (BioLegend). The presence of iNOS was detected using anti-iNOS antibody (polyclonal, Abcam, Cambridge, UK) and with FITC-conjugated secondary antibody (Vector Laboratories). Cell nuclei were stained with DAPI (Merck). Fluorescence was counted in 5 random fields (magn. 40×). Fluorescence imaging was performed with the confocal microscope LSM710 (Carl Zeiss Microscopy) and analysed with ImageJ 1.48v (NIH, Bethesda, MD, USA).

### Luminol-Based Chemiluminescence Assessment

Luminol chemiluminescence was assessed at four time points (0h, 6h, 12h, 24h). The mice were anesthetized with isoflurane (Piramal Critical Care, Hallbergmoos, Germany). Luminescence was measured using the IVIS Lumina system (PerkinElmer, Waltham, MA, USA), with a five-minute exposure time.

### TANs Magnetic Sorting

TANs were magnetically sorted using an Anti-Ly-6G MicroBeads UltraPure mouse kit, QuadroMACS Separator, and LS columns (Miltenyi Biotec, Bergisch Gladbach, Germany) according to the manufacturer’s instructions.

### TANs Intracellular Cytokines Assessment

Sorted TANs were lysed with IP buffer (Thermo Fisher Scientific) supplemented with protease inhibitors containing EDTA (Merck). The type and quantity of cytokines were assessed using the LEGENDplex™ Mouse Inflammation Panel (BioLegend) on a BD FACSCanto II flow cytometer (Becton Dickinson, Franklin Lakes, NJ, USA), according to the manufacturer’s instructions. The data were analyzed using LEGENDplex™ Data Analysis Software Version 8.0 (BioLegend).

### TANs in vitro and in vivo Cytotoxicity Assessment

For in vitro cytotoxicity analyses, 4T1 cells were placed in 96-well plates and incubated for 24 h. Sorted TANs were added at 10:1, 20:1, and 40:1 effector-to-target cell ratios. Cell viability was analyzed using an MTS assay (Promega, Madison, WI, USA) according to the manufacturer’s instructions. For in vivo cytotoxicity analyses, the following groups of mice were prepared: 1) 4T1+TAN cGAMP, 2) 4T1+TAN PBS, and 3) 4T1. Mice were injected subcutaneously with a mixture of 0.5×10^5^ sorted TANs (either from cGAMP- and PBS-treated tumors) with 0.5×10^5^ 4T1 cancer cells (1:1 proportion) in 100µL of PBS (groups 1 and 2) or with 0.5×10^5^ 4T1 cancer cells (group 3). The tumor volumes were determined using the following formula: volume=width^2^×length×0.52.

### Neutrophil Depletion Experiment

The mice with 4T1 tumors were divided into six groups: PBS, cGAMP, ISO, a-Ly6G, a-Ly6G+cGAMP, and ISO+cGAMP. Six days after cancer cell inoculation, mouse anti-Ly6G antibody or mouse isotype control antibody was administered by intraperitoneal injection every second day at a dose of 100 μg/mouse. cGAMP was administered nine days after cancer cell inoculation. Tumor volumes were determined using the formula: volume=width^2^×length×0.52. Depletion of Ly6G^+^ cells was confirmed by cytometric assessment of blood neutrophils. Blood was collected from the submandibular vein. The following antibodies (BioLegend) were used to assess neutrophil depletion: anti-CD11b (clone: M1/70, APC), anti-Ly6C (clone: HK1.4, PE-Cy7), and anti-Gr1 (clone: RB6-8C5, FITC). Dead cells were stained using DAPI (Merck). Gates dividing negative from positive cells were based on fluorescence minus one (FMO) control (BD FACSCanto II flow cytometer (Becton Dickinson)).

### Spontaneous 4T1 Lung Metastases Assessment

Murine 4T1 cells were subcutaneously injected. cGAMP/PBS was administered eight days after cancer cell inoculation. After the next four days, the mice were deeply anesthetized with intraperitoneal injection of ketamine (100 mg/kg) and xylazine (10 mg/kg). The mice were additionally protected using buprenorphine (0.1 mg/kg) and lidocaine (2 mg/kg). The primary tumors were excised surgically. The mice were left alive for 28 days after cancer cell inoculation. Tumor metastases were visualized by intratracheal injection of 15% India Ink solution. The number and size of lung metastases were manually calculated.

### Flow Cytometry Analysis of Immune Cells in the Lungs

Twelve hours after cGAMP/PBS administration, mouse lungs were collected and digested with collagenase type II solution (Gibco™, Thermo Fisher Scientific) supplemented with DNase I (Merck). Mononuclear cells and granulocytes were collected using Histopaque-1119 gradient centrifugation (Merck). The following antibodies (BioLegend) were used to assess immune cells subtypes: anti-CD11b (clone: M1/70, FITC), anti-Ly6G (clone: 1A8, PE, APC-Cy7), anti-CD69 (clone: H1.2F3, PE-Cy7), anti-CD54 (ICAM) (clone: YN1/1.7.4, APC) - neutrophils; anti-CD11b (clone: M1/70, PerCP-Cy5.5), anti-Ly6C (clone: HK1.4, PE) - monocytes; anti-CD11b (clone: M1/70, PerCP-Cy5.5), anti-F4/80 (clone: BM8, PE-Cy7), anti-CD206 (clone: C068C2, APC), anti-CD86 (clone: GL-1, BV510), anti-MHC II (clone: M5/114.15.2, FITC) - macrophages; anti-CD45 (clone: 30-F11, APC-Cy7), anti-CD49b (clone: DX5, PE), anti-NKp46 (clone: 29A1.4, PerCP-Cy5.5), anti-CD69 (clone: H1.2F3, BV510) - NK cells; anti-CD45 (clone: 30-F11, APC-Cy7), anti-CD8a (clone: 56–6.7, FITC), anti-CD4, anti-PD1 (clone: 29F.1A12, PE-Cy7), anti-CD69 (clone: H1.2F3, BV510) - T lymphocytes. Dead cells were stained using DAPI (Merck). Gates dividing negative from positive cells were based on the FMO control.

### Bone Marrow-Derived Macrophages (BMDM) Isolation

Bone marrow progenitors were flushed out with RPMI 1640 medium supplemented with 10% FBS (EURx) and 1% penicillin-streptomycin (Biowest) from the femurs and tibias of 8-week-old BALB/c mice. Cells were placed in a 24-well plate in RPMI 1640 medium supplemented with 20% FBS, 1% penicillin-streptomycin, and 10 ng/mL recombinant mouse macrophage colony-stimulating factor (M-CSF, BioLegend). Non-adherent cells were eliminated by washing with PBS. After seven days of incubation, mature macrophages (M0 phenotype) were used for the experiments. M2 macrophages were obtained by 24h cultivation with mouse recombinant IL-4 at a concentration of 10ng/mL (BioLegend).

### Co-Culture of BMDM with TANs

Sorted TANs were added to M0 or M2 BMDM cultures at 2:1 and 4:1 TANs to BMDM ratios. After 24h of co-incubation, cells were harvested and stained for flow cytometry. Phenotype of BMDM following TANs co-culture was assessed with the following antibodies (BioLegend): anti-CD11b (clone: M1/70), anti-F4/80 (clone: BM8), anti-CD206 (clone: C068C2), anti-CD86 (clone: GL-1), anti-Ly6G (clone: 1A8). Dead cells were stained using DAPI. Gates dividing negative from positive cells were based on the isotype control. Ly6G^+^ cells were excluded from the macrophage analyses.

### BMDM Treatment with cGAMP and Phenotype Assessment

M0 BMDM were treated with 5µM cGAMP for 24h. The harvested cells were incubated with the following antibodies (BioLegend): anti-CD11b (clone: M1/70), anti-F4/80 (clone: BM8), anti-CD206 (clone: C068C2), anti-CD86 (clone: GL-1), and anti-MHC II (clone: M5/114.15.2). Dead cells were stained using DAPI. In flow cytometry analyses (BD FACSCanto II), gates dividing negative from positive cells were based on an isotype control.

### Statistical Analysis

The results were analyzed using Statistica software version 12 (TIBCO Software Inc., StatSoft Poland, Krakow, Poland). The normality of the distribution was verified using the Shapiro–Wilk test. Homogeneity of variance was checked using Brown–Forsythe and/or Levene’s tests. Student’s *t*-test or Mann–Whitney *U*-test was used to compare two groups of variables. The Kruskal–Wallis test with Dunn’s multiple comparison post-hoc test or one-way analysis of variance with appropriate post-hoc test was used to compare more than two groups of variables. *p*-values < 0.05 were considered statistically significant. Data are shown as mean ± Standard Error of Mean (SEM).

## Results

### Targeting STING Triggers Oxidative Burst in vivo and Induces Changes in TANs Abundance, Activation, and Polarization

**Figure 1:**
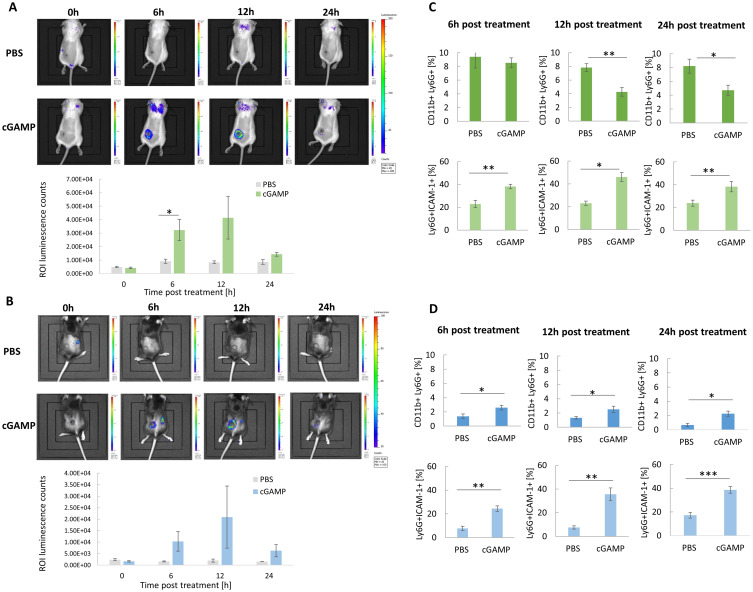
In vivo chemiluminescence imaging following STING agonist administration. (**A** and **B**) Mice with 4T1 (**A**) and B16-F10 (**B**) tumors were administered with luminol sodium salt at four time points after cGAMP treatment. The images were taken using the IVIS Lumina System, representative images from one independent experiment are shown. The ROI luminescence were counted with IVIS Lumina software, n=3. (**C** and **D**) Time course of TANs infiltration and polarization following cGAMP administration. The percentage of CD11b^+^Ly6G^+^ TANs and the N1-like Ly6G^+^ICAM-1^+^ TANs subpopulation in 4T1 (**C**) and B16-F10 (**D**) tumors. Tumors were collected at 3 time points: 6h, 12h, 24h. The data were acquired with flow cytometry. The statistical significance: (**A**) Student’s *t*-test *p<0.05. (**C**) CD11b^+^Ly6G^+^ 12h, 24h; Ly6G^+^ICAM-1^+^ 6h Student’s *t*-test *p<0.05; **p<0.01;; Ly6G^+^ICAM-1^+^ 12h, 24h Mann–Whitney *U*-test *p<0.05; **p<0.01. (**D**) CD11b^+^Ly6G^+^ 6h, 12h, 24h; Ly6G^+^ICAM-1^+^ 6h, Mann–Whitney *U*-test *p<0.05, **p<0.01; Ly6G^+^ICAM-1^+^ 12h, 24h Student’s *t*-test **p<0.01; ***p<0.001. Data from 2 independent experiments are shown, n=5, mean±SEM.

Luminol-based bioluminescence imaging was conducted to estimate the STING-agonist-induced oxidative burst. Analyses were performed after intratumoral cGAMP administration in 4T1 (Figure 1A) and B16-F10 tumor-bearing mice (Figure 1B). The administration of cGAMP resulted in the appearance of a high luminescence signal around the tumors 12 h post-treatment in both types of tumor models. In the case of 4T1 tumor-bearing mice, we observed additional signals at the location corresponding to the lungs. To confirm these results, we assessed the influence of STING targeting on TANs influx and the N1 TANs marker ICAM-1. The percentage of TANs in 4T1 tumors (Figure 1C) was approximately 8-fold higher compared to B16-F10 tumors (Figure 1D). In response to cGAMP administration in 4T1 tumors, 12 and 24 h post-injection, we observed an almost 2-fold decrease in the percentage of TANs. In both types of tumors, the highest increase in ICAM-1^+^ TANs was observed 12 h post cGAMP administration.

### STING Agonist Induces TANs Phenotype Switch

**Figure 2:**
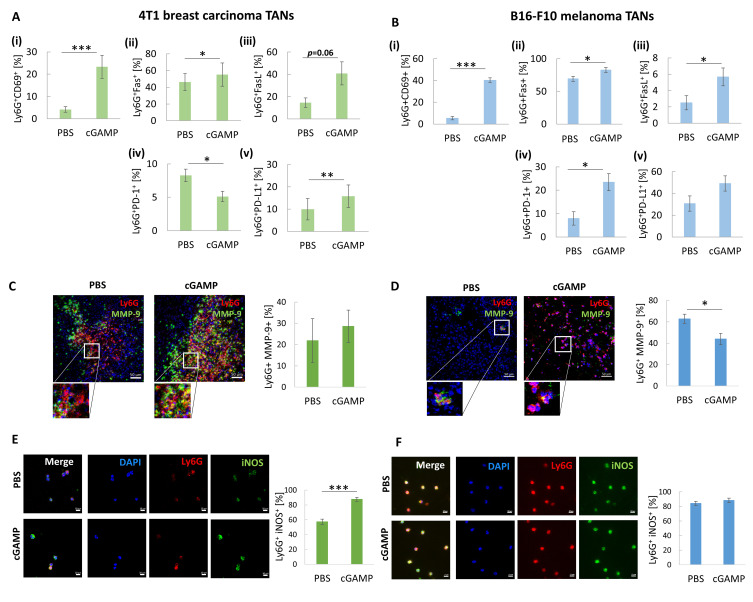
The analysis of cGAMP-induced TANs phenotype. (**A** and **B**) The percentage of the following CD11b^+^Ly6G^+^ subpopulation of TANs has been analysed 12 hours post cGAMP treatment: i) CD69^+^, ii) Fas^+^, iii) FasL^+^, iv) PD-1^+^, v) PD-L1^+^ in 4T1 (**A**) and B16-F10 (**B**) tumors. The statistical significance: (**A**) ii), iv), v), (**B**) i), ii), iv) Student’s *t*-test *p<0.05; **p<0.01; ***p<0.001; (**A**) i), iii) (**B**) iii) Mann–Whitney *U*-test *p<0.05; ***p<0.001, n=5, mean±SEM. (**C** and **D**) Tumor sections were stained with appropriate primary antibodies to visualize MMP-9 (FITC, green) presence in TANs (Ly6G, Alexa Fluor 594, red). Nuclei were stained with DAPI (blue). The cells were visualized using confocal microscope, 20x magnification. Representative images are shown. The percentage of MMP9^+^ TANs was assessed with the use of flow cytometry analysis, based on isotype controls, Mann–Whitney *U*-test *p<0.05, n=5, mean±SEM. (**E** and **F**) Sorted TANs were cytospined and stained with primary antibodies: anti-Ly6G (Alexa Fluor 594, red) and anti-iNOS (FITC, green). Nuclei were stained with DAPI (blue). The iNOS^+^ TANs were visualized using a confocal microscope, 40x magnification. Percentage of iNOS^+^ TANs was calculated using ImageJ software, Mann–Whitney *U*-test ***p<0.001, n=4. Data from 2 independent experiments are shown, mean±SEM.

In both tumor models, an increase in the percentage of CD69^+^, Fas^+^, FasL^+^ TANs was observed 12 h after cGAMP administration (Figure 2A (i), (ii), (iii); B (i), (ii), (iii)). Depending on the tumor model, we observed a decrease (4T1 TANs) or an increase (B16-F10 TANs) in PD-1 expression (Figure 2A (iv); B (iv)). Simultaneously, an increase in the percentage of PD-L1^+^ TANs was observed (Figure 2A (v); B (v)). In both types of tumors, immunohistochemical assessment revealed matrix metalloproteinase 9 (MMP-9) positive cells. In 4T1 tumors, we noticed a slight increase in MMP-9 expression in TANs derived from cGAMP-treated mice (Figure 2C). In B16-F10 tumors, a decrease in MMP-9^+^ TANs was observed following cGAMP administration, from 63% in control cells to 43% (Figure 2D). To estimate the percentage of iNOS^+^ TANs, magnetically sorted cells were subjected to immunocytochemical staining. We noticed a significant increase in iNOS expression in TANs from cGAMP-exposed 4T1 tumors compared to that in control TANs. The percentage of Ly6G^+^iNOS^+^ cells increased from 57 to 88% after STING agonist administration (Figure 2E). For B16-F10-derived TANs, we did not observe a difference in the percentage of Ly6G^+^iNOS^+^ cells between cGAMP-exposed and control tumors. In both groups, more than 80% of the TANs were iNOS^+^ (Figure 2F).

### Targeting STING Triggers TANs to Acquire an N1-Like Signature of Intracellular Cytokines

**Figure 3:**
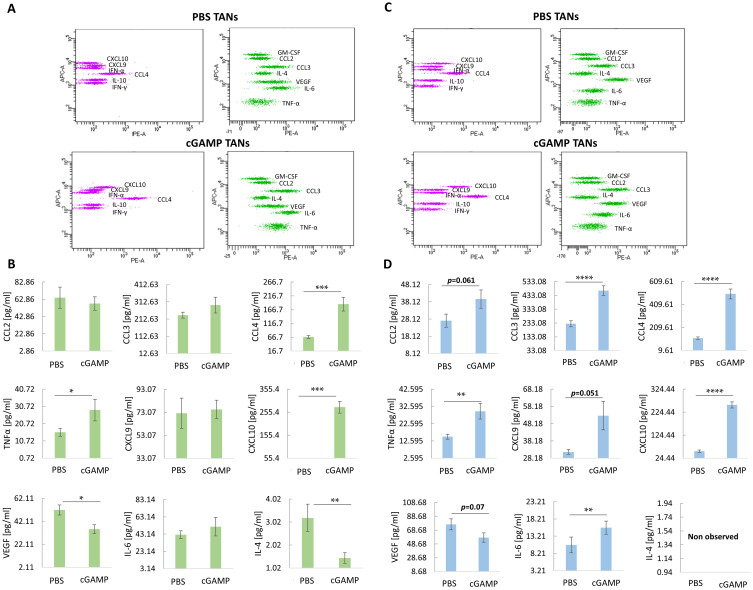
Intracellular cytokines profile of TANs following cGAMP treatment. (**A** and **C**) Representative flow cytometry graph from cytokines analysis of 4T1 tumors-derived TANs (**A**) and B16-F10 tumors-derived TANs (**C**) following intratumoral STING agonist administration. (**B**) Panel of 4T1 tumor-derived TANs cytokines. The statistical significance: Mann–Whitney *U*-test *p<0.05; **p<0.01; ***p<0.001. (**D**) Panel of B16-F10 tumor-derived TANs cytokines. The statistical significance: CCL3 chart – Student’s *t*-test ****p<0.0001, the other charts – Mann–Whitney *U*-test **p<0.01; ****p<0.0001. Data from 3 independent experiments are shown, n=4, mean±SEM.

Using a multiplex immunoassay, we characterized the TANs cytokine repertoire post in vivo cGAMP treatment. For each sample, the same number of TANs was lysed, and cytokine levels were calculated based on the assay standard curve (Figure 3A-D). Following cGAMP treatment, we observed increased levels of classical cytokines for N1 TANs (CCL3, TNFα, and IL-6) with a simultaneous decrease in cytokine levels typical for N2 TANs (VEGF). In TANs derived from cGAMP-treated 4T1 tumors, CCL3, CCL4, and TNFα levels were 1.25-, 2.7-, and 1.8-fold higher, respectively (Figure 3B). cGAMP-treated 4T1 tumor-derived TANs showed a 7-fold increase in CXCL10 and 1.5- and 2-fold decreases in VEGF and IL-4, respectively (Figure 3B). In cGAMP-treated B16-F10 tumor-derived TANs, the levels of CCL3, CCL4, and TNFα increased 2-, 4.3-, and 2-fold, respectively (Figure 3D). TANs from cGAMP-injected B16-F10 tumors were characterized by 1.7-, 4.5-, and 1.4-fold increases in CXCL9, CXCL10, and IL-6 levels, respectively, and a 1.3-fold decrease in VEGF levels (Figure 3D).

### STING Agonist Unleashes TANs Cytotoxic Potential

**Figure 4:**
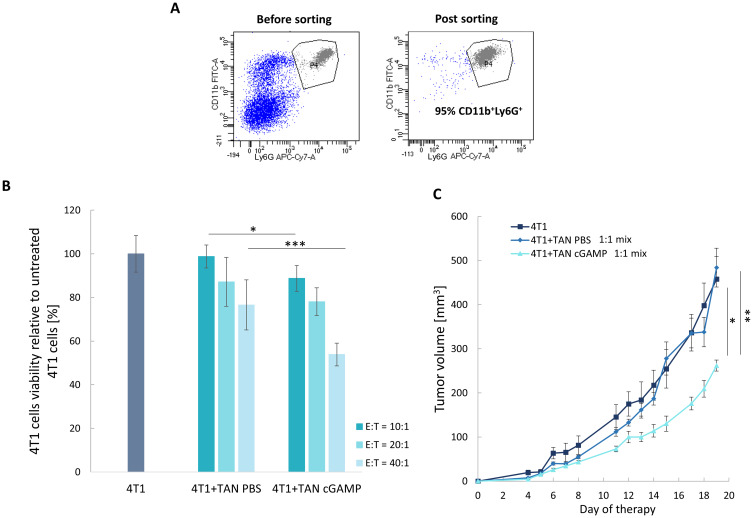
STING agonist–induced TANs cytotoxicity toward 4T1 breast cancer. TANs were sorted from tumors treated with cGAMP or PBS. (**A**) Sorting purity was assessed before each experiment. (**B**) TANs cytotoxicity toward 4T1 cancer cells in vitro. Cells were cultivated in three ratios of TANs (E, effector) to 4T1 cells (T, target). The statistical significance: ANOVA with Tukey’s HSD post-hoc test *p<0.05; ***p<0.001; n=3, data from 3 independent experiments are shown, mean±SEM. Additional statistical significance not shown on the graph: 4T1 vs 4T1+TAN PBS (40:1) ***p<0.001; 4T1 vs 4T1+TAN cGAMP (10:1) *p<0.05; 4T1 vs 4T1+TAN cGAMP (20:1) **p<0.01; 4T1 vs 4T1+TAN cGAMP (40:1) ***p<0.001. (**C**) TANs-induced 4T1 tumor growth inhibition in vivo. BALB/c mice were subcutaneously injected with the mixture of 4T1 and TANs (1:1 ratio). Mice in the 4T1 group were inoculated with cancer cells only. The statistical significance: ANOVA with Tukey’s HSD post-hoc test *p<0.05; **p<0.01. Representative data from 1 of 2 independent experiments are shown, n=4, mean±SEM.

The cytotoxicity of TANs derived from cGAMP-treated 4T1 tumors was tested in vitro and in vivo. Due to a significantly lower number of TANs in both control and cGAMP-treated B16-F10 tumors, the experiments assessing TANs killing potential were performed only on the 4T1 model. The population of TANs was sorted with a purity of approximately 95% (Figure 4A). TANs from cGAMP-treated tumors decreased 4T1 cancer cells viability to 54%, whereas TANs from control tumors reduced cancer cells viability to 77% (40:1 – effector:target cells) (Figure 4B). To confirm the potential of the STING agonist to elevate TANs cytotoxic functions, mice were subcutaneously injected with a mixture of 4T1 cancer cells and TANs sorted from cGAMP-treated or control tumors (1:1 – TANs:4T1). The tumor volume in the group receiving the mixture of 4T1 cells with TANs sorted from cGAMP-treated mice was 1.7-fold smaller than that in control 4T1 tumors and 1.8-fold smaller in comparison to tumors of mice that received a mixture of 4T1 cells with control TANs (Figure 4C).

### Neutrophil Abolition May Have a Detrimental Effect During STING Targeting Therapy

**Figure 5:**
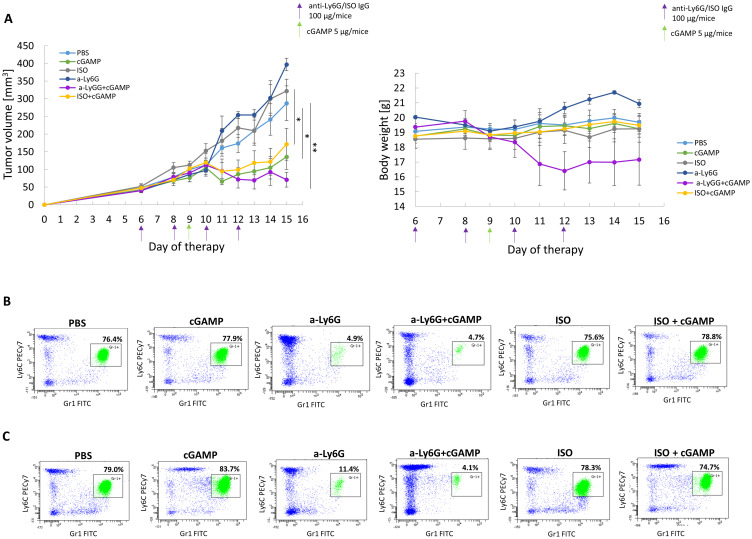
The impact of anti-Ly6G antibody and STING agonist treatment on mice with 4T1 tumors. (**A**) The effect of STING agonist and Ly6G blocking antibody treatment on 4T1 tumor volume (left chart) and mice body weight (right chart). ANOVA with post-hoc Fisher’s LSD test *p<0.05; **p<0.01. Additional statistical significance not shown on the left graph: ISO+cGAMP vs a-Ly6G **p<0.01; cGAMP vs ISO**p<0.01; cGAMP vs a-Ly6G **p<0.01; a-Ly6G+cGAMP vs ISO **p<0.01; a-Ly6G+cGAMP vs a-Ly6G **p<0.01. Data from 2 independent experiments are shown, n=3, mean±SEM. (**B** and **C**) The representative flow cytometry dot plots of blood Ly6G^+^ neutrophils (Ly6C^+^Gr-1^+^) assessment one day before (**B**) and one day after (**C**) cGAMP treatment.

To assess the impact of neutrophils in STING-activating therapy, we performed pilot experiments with neutrophil depletion (Figure 5A). The effectiveness of the anti-Ly6G antibody was verified by the assessment of blood neutrophils (Ly6C^+^Gr-1^+^ cells). Mice receiving anti-Ly6G antibody showed a decrease in the Ly6C^+^Gr1^+^ population, corresponding to Ly6G^+^ neutrophils, and an increase in the Ly6C^+^Gr1^−^ population, indicating masking of neutrophils by the antibody (Figure 5B and C). Masking neutrophils with anti-Ly6G antibody did not affect the size of 4T1 tumors or mouse weight. In mice that received cGAMP, significant inhibition of tumor growth was observed. In mice with neutrophil depletion, the STING agonist inhibited tumor growth; however, it resulted in an extreme decrease in the body weight of mice and deterioration of the condition of several individuals (Figure 5A).

### STING Agonist Activates Lung Immune Cells and Decreases Metastasis Potential

**Figure 6:**
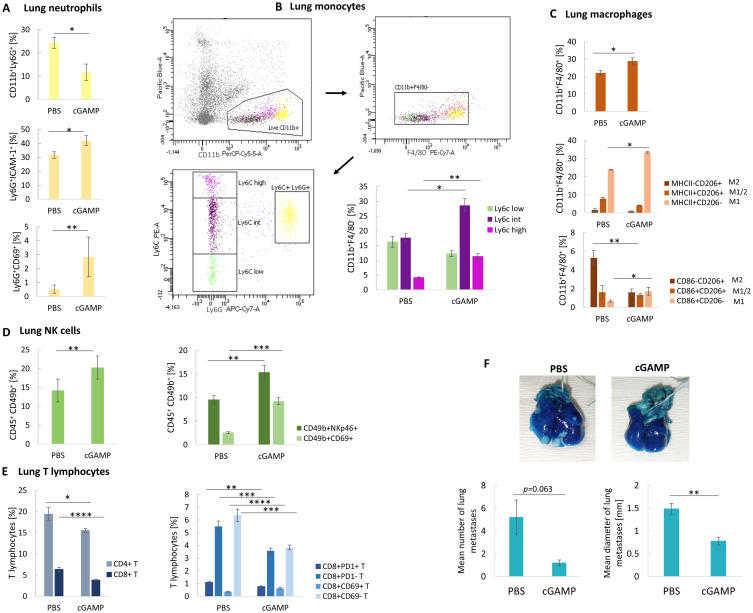
The phenotype switch of lung immune cells after STING agonist intratumoral administration. (**A**) The percentage of CD11b^+^Ly6G^+^ neutrophils and N1-like Ly6G^+^ICAM^+^ and Ly6G^+^CD69^+^ neutrophil subpopulations. CD11b^+^Ly6G^+^, Ly6G^+^ICAM^+^ Student’s *t*-test *p<0.05; Ly6G^+^CD69^+^ Mann–Whitney *U*-test **p<0.01. (**B**) The flow cytometry graph showing gating strategy for lung monocytes assessment (live myeloid cells CD11b^+^, exclusion of F4/80 macrophages, exclusion of Ly6G neutrophils), and the percentage of CD11b^+^F4/80^−^ monocyte subpopulations. Ly6C^int^ Student’s *t*-test*p<0.05; Ly6C^high^ Mann–Whitney *U*-test **p<0.01. (**C**) The percentage of CD11b^+^F4/80^+^ macrophages, and their particular subpopulations. CD11b^+^F4/80^+^, MHCII^+^CD206^−^ Student’s *t*-test *p<0.05; CD86^+^CD206^−^, CD86^−^CD206^+^ Mann–Whitney *U*-test *p<0.05; **p<0.01. (**D**) The percentage of CD45^+^CD49b^+^ NK cells, and their particular subpopulations. CD45^+^CD49b^+^ Student’s *t*-test **p<0.01; CD49b^+^NKp46^+^ Student’s *t*-test **p<0.01; CD49b^+^CD69^+^ Mann–Whitney *U*-test ***p<0.001. (**E**) The percentage of CD8^+^ and CD4^+^ T lymphocytes, and the CD8^+^ T lymphocytes subpopulations. CD4^+^ and CD8^+^ T lymphocytes, Mann–Whitney *U*-test *p<0.05, ****p<0.0001; CD8^+^CD69^+^ T, CD8^+^CD69^−^ T, CD8^+^PD-1^+^ T, CD8^+^PD-1^−^ T Student’s *t*-test **p<0.01, ***p<0.001, ****p<0.0001. Data from 1-independent experiments are shown, n=5, mean±SEM. The effect of targeting STING on spontaneous 4T1 lung metastasis. (**F**) Representative photographs of lungs stained with India Ink to visualize the metastases. Mean number (left chart) and diameter (right chart) of lung metastases following cGAMP treatment. The statistical significance: Mann–Whitney *U*-test, **p<0.01. Data from 1 independent experiment are shown, n=5, mean±SEM.

The STING agonist induced a 2-fold decrease in the percentage of neutrophils with a simultaneous switch of their phenotype toward an antitumor one; an increase in ICAM-1^+^ and CD69^+^ neutrophil subpopulation was observed (Figure 6A). Further analyses indicated a 1.6-fold increase in Ly6C^int^ and a 2.7-fold increase in proinflammatory Ly6C^hi^ monocytes (Figure 6B). Following cGAMP treatment, lung macrophages were polarized toward the M1 phenotype, elevated number of MHCII^+^CD206^−^ and CD86^+^CD206^−^ cells (1.4- and 2.6-fold, respectively), with a simultaneous decrease in the M2 macrophages number (MHCII^−^CD206^+^ and CD86^−^CD206^+^) (Figure 6C). We observed infiltration and activation of NK cells after cGAMP treatment (CD49b^+^NKp46^+^: 1.6-fold increase; CD49b^+^CD69^+^: 3.6-fold increase) (Figure 6D) and a decrease in CD4^+^ and CD8^+^ T lymphocytes. Analyses of the CD8^+^ T cells phenotype showed a decrease in the exhausted (CD8^+^PD-1^+^) and a slight elevation in the activated (CD8^+^CD69^+^) T lymphocytes (Figure 6E). Implementation of the 4T1 model of spontaneous lung metastasis revealed a 4.3-fold decrease in the mean number and a 1.9-fold decrease in the mean diameter of the lung metastases (Figure 6F).

### STING Agonist-Polarized TANs Have the Potential to Switch Macrophage Phenotype

**Figure 7:**
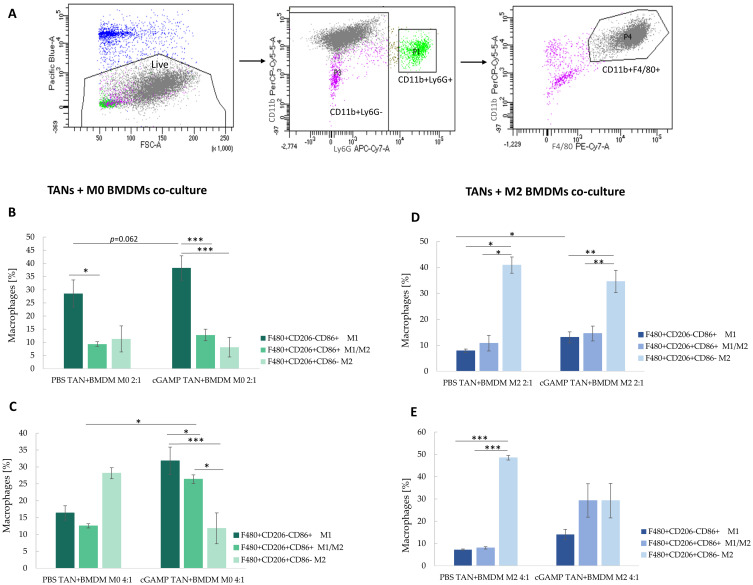
The phenotype of BMDM after co-culture with TANs. The upper panel shows a gating strategy to exclude CD11b^+^Ly6G^+^ cells from macrophages analysis. (**A**) The unstimulated BMDM cells were defined as M0 (**B** and **C**), IL-4-stimulated BMDM as M2 cells (**D** and **E**). The co-cultures of TANs and BMDM were established in ratios equal 2:1 (**B** and **D**) and 4:1 (**C** and **E**). BMDM phenotype was analysed with flow cytometry 24h post-co-culture. The statistical significance: (**B**) for two groups comparison: Student’s *t*-test *p<0.05; **p<0.01; ***p<0.001; for three groups comparison among PBS TAN+BMDM: Kruskal–Wallis test with Dunn’s multiple comparison post-hoc test *p<0.05; for three groups comparison among cGAMP TAN+BMDM: ANOVA with Tukey’s HSD post-hoc test ***p<0.001; (**C**) for two groups comparison Mann–Whitney *U*-test *p<0.05; for three groups comparison ANOVA with Tukey’s HSD post-hoc test *p<0.05; ***p<0.001; (**D**) for two groups comparison: Student’s *t*-test *p<0.05; for three groups comparison among PBS TAN+BMDM group: Kruskal–Wallis test with Dunn’s multiple comparison post-hoc test *p<0.05; for three groups comparison among cGAMP TAN+BMDM group: ANOVA with Tukey’s HSD post-hoc test **p<0.01; (**E**) for three groups comparison: ANOVA with Tukey’s HSD post-hoc test ***p<0.001. Data from 2 independent experiments are shown, n=3, mean±SEM.

Flow cytometry assessment with a proper gating strategy enables analysis of BMDM phenotype following direct co-cultivation with sorted TANs (Figure 7A). STING agonist-activated TANs exhibited a higher potential to polarize M0 BMDM into M1 cells than the control TANs (Figure 7B). The effect was more pronounced following an increase of TANs to BMDM ratio toward 4:1. Importantly, control TANs increased the percentage of M2 (CD206^+^CD86^−^) cells by up to 28% of the total analyzed macrophages, which constituted a 2.4-fold higher percentage of M2 cells compared to co-culture with TANs from cGAMP-treated tumors (Figure 7C). To mimic the tumor microenvironment, we assessed the potential of in vivo STING-activated TANs to re-polarize M2 macrophages. In vivo cGAMP-induced TANs co-cultured in a ratio of 2:1 with IL-4-polarized M2 BMDM confirmed the potential of STING agonist-activated TANs to shift M2 markers toward M1-like ones (Figure 7D). In the experiments where the TANs:BMDM M2 ratio was 4:1, we did not observe an increase in the percentage of M1 macrophages compared to the 2:1 ratio; however, we noted a decrease in the number of M2 macrophages following co-culture with TANs derived from cGAMP-treated tumors (Figure 7E). The phenotypes of M0 and M2 BMDM were also analyzed (Figure S1A and B). We additionally analyzed the phenotype of BALB/c-derived BMDM after direct treatment with STING agonist (Figure S2). The changes of BMDM phenotype toward M1 were observed following cGAMP treatment.

## Discussion

Currently, numerous efforts are underway to develop safe, effective, and personalized therapeutic strategies for cancer immunotherapy. Based on the tumor immunophenotypes, therapeutic approaches include normalization strategies of stromal cells (eg, targeting cancer-associated fibroblasts), immunity (eg, application of immune checkpoint inhibitors and adoptive cellular therapy), and tumor cells (eg, induced expression of antigens on tumor cells).^26^ Among them, targeting the STING pathway engages the host immune system to boost systemic anticancer immune response. STING activation stimulates the production of a wide range of proinflammatory cytokines and type I IFNs, triggering an organized immune reaction.^27^ Among the cells contributing to this response, neutrophils seem to be undervalued. Because targeting STING induces IFN-β production, a well-accepted factor for TANs repolarization,^13^,^17^ in the current study, we verified if STING agonist enables polarization of TANs into N1 cells, revealing their anticancer potential.

Previously, we and others have shown that particular types of tumors and cancer cell lines characterize different STING expression, which is associated with prognosis and therapy outcomes.^28–30^ Based on this, we assessed the STING agonist-induced polarization of the TANs phenotype in two murine tumor models with diversified STING status – STING^high^ B16-F10 melanoma and STING^low^ 4T1 breast carcinoma tumors.^24^,^28^ Initially, to indirectly assess the time point with increased neutrophil activity, we used luminol-based in vivo imaging technology. In both tumor models, we detected strong luminescence signals around the tumors occurring twelve hours after targeting STING. Its appearance confirms myeloperoxidase-dependent oxidative burst, which leads to rapid production of high levels of ROS and might be connected with the activity of both macrophages and neutrophils.^31^ Our analyses revealed that in 4T1 STING^low^ tumors, cGAMP treatment decreased the percentage of TANs residing within tumors. In contrast, in the case of STING^high^ B16-F10 tumors, in which TANs constitute a small population of immune cells, we noted an influx of TANs. In recent years, TANs have been recognized as a population of cells whose phenotype may undergo polarization. In our study, STING activation led to increased percentages of ICAM-1^+^ N1-like TANs in both tumor models. The presence of ICAM-1 is known to enhance neutrophil effector functions and ROS generation.^32^ In both types of tumors, further analysis showed that STING agonist-induced increase in the level of TANs activation and degranulation marker (CD69) and other N1 markers (Fas, FasL), which, besides promoting cancer cells death, may also enhance cytotoxic T lymphocyte activity.^15^,^33–35^ Additionally, following STING activation, we noted increased PD-L1 expression in TANs derived from both types of tumors. Importantly, due to possible suppression of cytotoxic T cells mediated by PD-L1, its presence on TANs is considered a feature of pro-tumor cells.^36^,^37^ On the other hand, neutrophils were shown to upregulate PD-L1 expression toward the resolution of inflammation.^38^,^39^ Similar to our previous research, where the STING agonist was shown to decrease the presence of PD-1 on CD8^+^ T lymphocytes in 4T1 tumors, while increasing its presence on T cells in B16-F10 tumors,^28^ targeting STING also decreased the percentage of PD-1^+^ TANs in 4T1 tumors and increased it in B16-F10 tumors. STING agonists, while having a great impact on switching tumors’ immunophenotype toward a hot one, may increase PD-1 expression in some tumors. Thus, the combination of STING targeting therapies with the blockage of the PD-1/PD-L1 axis is of great importance to counteract the immunosuppressive effects of STING activation and to augment therapy outcomes.^39–41^ In favor of such an approach, blocking the PD-L1/PD-1 axis has been shown to increase neutrophil antitumor cytotoxicity and is considered the gold standard for combination therapies in cancer treatment.^38^ One of the fundamental roles of neutrophils is the maintenance of tissue homeostasis. To achieve this, they act through multiple mechanisms, such as the ROS2- and iNOS-dependent generation of reactive oxygen and nitrogen species. Although the products of ROS2 and iNOS may lead to inflammation and subsequent carcinogenesis, their manifestation is not currently recognized as a strict N2 pro-tumor TANs markers. Since TANs may directly kill cancer cells through the action of iNOS and ROS2, these characteristics are considered as N1 TANs markers.^42–44^ In the current study, following cGAMP treatment, we observed increased iNOS expression in 4T1-derived TANs. In the case of B16-F10-derived TANs, we did not observe a STING agonist-induced increase in iNOS expression, which might be caused by its initially high expression noted in control TANs.

TANs may exert their functions through the release of various cytokines. Additionally, their composition may differentiate from TAN subtypes.^45^ Evaluation of TANs intracellular cytokines following treatment with STING agonist demonstrated a switch of cytokine repertoire toward that typical for N1 TANs. We noticed an increase in TNFα, CXCL-10, CCL3, CCL4, and IL-6 and a decrease in VEGF content. A similar set of N1-specific cytokines was observed by Mihaila et al in mouse bone marrow-derived neutrophils following in vitro polarization with LPS+IFN-γ.^46^ Importantly, the increased presence of IL-6 was previously connected with the features of N2-like TANs.^18^ Considering the current data, it is worth noting that targeting STING may lead to the activation of both IRF3 and NF-κB pathways, promoting the expression of type I IFNs and proinflammatory cytokines, respectively. Among them, TNFα, IL−1β, and IL-6 are known to be upregulated following targeting of STING.^47^ Human neutrophils have low mRNA expression of TMEM173 (STING); however, its measurable level suggests the possible translation into protein.^48^,^49^ Thus, in the current study, increased levels of particular cytokines may be the result of the direct impact of cGAMP on TANs.

STING agonists were shown to drive tumor infiltration with innate immune cells, providing an anticancer response.^50^ In the study assessing the role of neutrophils in STING-activating therapy, Nagata et al showed enhanced cytotoxicity of spleen-derived neutrophils following in vitro IFN-β treatment.^51^ In the other work, Benguigui et al indicated that induction of 4T1 tumor-intrinsic STING activity induces Ly6E^hi^ neutrophils, which sensitize resistant 4T1 tumors to anti-PD1 therapy. Moreover, the study showed that IFNα/γ-induced Ly6E^hi^ neutrophils participate in the activation of effector T cells.^8^ To the best of our knowledge, no study has revealed increased TANs’ killing capabilities directly following in vivo STING activation. According to our hypothesis, we have shown that targeting STING leads to activation of TANs, enabling the manifestation of their cytotoxic potential against 4T1 cancer cells.

TANs are known to support tumor growth and progression during the late stages of tumor development.^52^ Thus, using an antibody directed against Ly6G cells, we aimed to verify the role of neutrophils in STING-targeted therapy. Both in the group treated with STING agonist and in the combination group that received STING agonist and anti-Ly6G antibody, similar tumor growth inhibition was observed. Although the results of neutrophil neutralization did not show that they are the key cells for STING agonist-induced 4T1 tumor growth inhibition, our findings revealed the importance of their presence for the maintenance of the health conditions of mice undergoing STING-activating therapy. The observed lack of increased tumor growth following STING activation in mice treated with the anti-Ly6G antibody might be partially affected by the blocking of both cGAMP-induced TANs with antitumor functions and tumor-supporting resident TANs. Importantly, individuals treated with both anti-Ly6G antibody and STING agonist dramatically lost weight and manifested deterioration of their health condition. Recently, Knox et al revealed the importance of controlled STING expression in transgenic mice, with repressed STING expression in neutrophils. Following forced STING expression, scientists noticed inhibited neutrophil maturation, viability, and signs of systemic inflammatory disease.^53^ Based on these preliminary results and our data, it seems to be of great importance to verify the possible implication of STING-activating therapies for cancer patients with chemotherapy-induced neutropenia.

The potential of STING activation to inhibit the process of metastasis has been revealed in several studies.^54–56^ Previously, we have shown the impact of local targeting of STING on systemic changes among blood immune cells, hematological properties, and serum cytokines. We observed a decrease in the number of white blood cells, including granulocytes, and increased production of type I IFNs and CXC chemokines in the blood of 4T1 tumor-bearing mice following intratumoral cGAMP administration.^24^ Considering these results, we verified the capability of locally targeting STING to transform the lung immune cells phenotype toward an activated, antitumor phenotype. Similar to the observations made in tumors, following cGAMP treatment, we noticed a decrease in lung-residing neutrophils, with simultaneous polarization of their state toward N1-like activated cells, characterized by increased ICAM-1 and CD69 expression. Further analysis of lung myeloid cells revealed a cGAMP-induced infiltration and polarization of lung macrophages toward proinflammatory anticancer M1 cells. Previously, Hagerling et al found that reprogrammed through IFN-γ–producing monocytes, lung neutrophils participate in the response against metastatic breast cancer.^57^ These results may support the hypothesis that the decrease in metastasis burden following STING activation is partially dependent on the transformation of particular myeloid cell subtypes. Recently, NK cells have been recognized as important contributors to STING-dependent antitumor and antimetastatic responses.^50^,^58^,^59^ Following STING targeting, we noticed a significant influx of NK cells toward the lungs, which was accompanied by their activation. To complete the analysis of lung immune cells following cGAMP treatment, we investigated the population of T lymphocytes. Similar to our previous research,^28^ here we observed a decrease in lung CD4^+^ and CD8^+^ T lymphocytes following STING targeting. Taken together, these results indicate that STING activation induces a systemic immune response, enabling the activation and switching of the lung immune cells phenotype, consequently inhibiting the metastasis process.

The STING pathway is involved in innate immunity, which is commonly dysregulated during cancer development. Therefore, it is important to consider mutual interactions among particular immune cell subtypes following targeting of STING.^24^,^60^,^61^ In the current study, we assessed the impact of in vivo cGAMP-stimulated TANs on macrophage phenotype. The results from the direct co-culture of TANs derived from cGAMP-treated tumors with bone marrow-derived macrophages (BMDM) revealed an increased transition of the BMDM phenotype toward antitumor M1 in comparison to co-culture conducted with control TANs. A similar effect was observed in the co-culture of TANs with M2-polarized BMDM. Based on these results, we speculated that TANs may be partially responsible for STING agonist-induced M1 macrophage polarization.

The findings of the current research focused on the direct polarization of TANs by STING activation and the resulting distal immune remodeling; however, there are a few limitations of the study. One of the issues that remains to be explored is the direct impact of the STING agonist within TANs. The other open questions that appeared during the study concern the mechanistic explanation for elevated PD-L1 on N1-like TANs, and the specific causes of toxicity following neutrophil depletion.

Taken together, our preclinical study revealed that STING agonist induces conversion of the TANs phenotype toward antitumor N1-like, which unleashes TANs cytotoxic potential. Moreover, we showed the potential abscopal effect of cGAMP and the ability of STING agonist-activated TANs to shift the macrophages’ phenotype toward the M1 antitumor state.

## Conclusions

Targeting STING emerges as a potent and promising anti-cancer strategy. Simultaneously, its chronic activation can create an immunosuppressive tumor microenvironment, hindering efficient anti-cancer immunity. The obtained data imply the possibility of STING agonist-induced remodelling of the tumor microenvironment by modulating innate immunity. STING agonist enables polarization of the TANs toward the anticancer N1 subtype. Targeting STING shifts the tumor microenvironment from immunosuppressive toward immunostimulatory, which is accompanied by activation of TANs. Such changes inhibited the metastasis process and enabled the acquisition of the M1 phenotype by macrophages.

## Data Availability

All data relevant to the study were included in the article or uploaded as supplemental online information. Any additional information required to reanalyze the data reported in this study is available from the lead contact upon request. The datasets used and analyzed during the current study are available from the corresponding author (Alina Drzyzga) upon reasonable request.
